# The de-ubiquitylating enzymes USP26 and USP37 regulate homologous recombination by counteracting RAP80

**DOI:** 10.1093/nar/gkv1480

**Published:** 2015-12-15

**Authors:** Dimitris Typas, Martijn S. Luijsterburg, Wouter W. Wiegant, Michaela Diakatou, Angela Helfricht, Peter E. Thijssen, Bram van den Broek, Leon H. Mullenders, Haico van Attikum

**Affiliations:** 1 Department of Human Genetics, Leiden University Medical Center, Einthovenweg 20, 2333 ZC, Leiden, The Netherlands; 2 Biophysics of Cell Signaling, The Netherlands Cancer Institute, Plesmanlaan 121, 1066 CX, Amsterdam, The Netherlands

*Nucl. Acids Res*. 43 (14): 6919–6933. doi: 10.1093/nar/gkv613

The name of one of the authors has been misspelled in the article and should be changed from Bram van **de** Broek to Bram van **den** Broek.

The authors apologise to the readers for the inconvenience caused.

